# The p21‐activated kinase 2 (PAK2), but not PAK1, regulates contraction‐stimulated skeletal muscle glucose transport

**DOI:** 10.14814/phy2.14460

**Published:** 2020-06-29

**Authors:** Lisbeth L. V. Møller, Ida L. Nielsen, Jonas R. Knudsen, Nicoline R. Andersen, Thomas E. Jensen, Lykke Sylow, Erik A. Richter

**Affiliations:** ^1^ Section of Molecular Physiology Department of Nutrition, Exercise and Sports Faculty of Science University of Copenhagen Copenhagen Denmark

**Keywords:** contraction, glucose uptake, p21‐activated kinase, skeletal muscle

## Abstract

**Aim:**

Muscle contraction stimulates skeletal muscle glucose transport. Since it occurs independently of insulin, it is an important alternative pathway to increase glucose transport in insulin‐resistant states, but the intracellular signaling mechanisms are not fully understood. Muscle contraction activates group I p21‐activated kinases (PAKs) in mouse and human skeletal muscle. PAK1 and PAK2 are downstream targets of Rac1, which is a key regulator of contraction‐stimulated glucose transport. Thus, PAK1 and PAK2 could be downstream effectors of Rac1 in contraction‐stimulated glucose transport. The current study aimed to test the hypothesis that PAK1 and/or PAK2 regulate contraction‐induced glucose transport.

**Methods:**

Glucose transport was measured in isolated soleus and extensor digitorum longus (EDL) mouse skeletal muscle incubated either in the presence or absence of a pharmacological inhibitor (IPA‐3) of group I PAKs or originating from whole‐body PAK1 knockout, muscle‐specific PAK2 knockout or double whole‐body PAK1 and muscle‐specific PAK2 knockout mice.

**Results:**

IPA‐3 attenuated (−22%) the increase in glucose transport in response to electrically stimulated contractions in soleus and EDL muscle. PAK1 was dispensable for contraction‐stimulated glucose transport in both soleus and EDL muscle. Lack of PAK2, either alone (−13%) or in combination with PAK1 (−14%), partly reduced contraction‐stimulated glucose transport compared to control littermates in EDL, but not soleus muscle.

**Conclusion:**

Contraction‐stimulated glucose transport in isolated glycolytic mouse EDL muscle is partly dependent on PAK2, but not PAK1.

## Graphical abstract

In this study, we show that contraction‐stimulated glucose transport in isolated mouse skeletal muscle is unaffected by whole‐body knockout of PAK1. On the contrary, muscle‐specific knockout of PAK2 either alone or in combination with whole‐body PAK1 knockout partially reduces contraction‐stimulated glucose transport in glycolytic EDL muscle. These data suggest that contraction‐stimulated glucose transport in isolated glycolytic mouse EDL muscle is partly dependent on PAK2, but not PAK1.

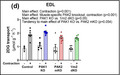

## 1 INTRODUCTION

Muscle contraction increases skeletal muscle glucose uptake independently of insulin (Douen et al., 1990; Hansen, Gulve, & Holloszy, 1994; Lund, Holman, Schmitz, and Pedersen 1995; Ploug, Galbo, & Richter, 1984; Ploug, van Deurs, Ai, Cushman, & Ralston, 1998; Wallberg‐Henriksson & Holloszy, 1985). Accordingly, muscle contraction increases glucose uptake similarly in both insulin‐sensitive and insulin‐resistant skeletal muscle (Kennedy et al., 1999; Martin, Katz, & Wahren, 1995; Richter & Hargreaves, 2013). Additionally, insulin sensitivity is improved after cessation of muscle contraction (Mikines, Sonne, Farrell, Tronier, & Galbo, 1988; Richter, Garetto, Goodman, & Ruderman, 1982; Richter, Mikines, Galbo, & Kiens, 1989; Wojtaszewski, Hansen, Kiens, & Richter, 1997), making muscle contraction during acute exercise a nonpharmacological treatment for insulin resistance (Sylow & Richter, 2019). However, while muscle contraction is known to promote the translocation of the glucose transporter (GLUT)‐4 to the plasma membrane, which facilitates glucose entry into the muscle, the intracellular signaling regulating this process is incompletely understood.

Upon muscle contraction, multiple intracellular signaling pathways are activated that promote GLUT4 translocation and a subsequent increase in muscle glucose uptake. Redundant Ca^2^^+^‐dependent signaling, metabolic stress signaling, and mechanical stress signaling are proposed to regulate distinct steps important for glucose transport in response to muscle contraction (Sylow, Kleinert, Richter, & Jensen, 2017). The group I p21‐activated kinase (PAK)‐1 and PAK2 are activated in response to electrical pulse stimulation in C2C12 myotubes (Hu et al., 2018; Yue et al., 2019) and muscle contraction/acute exercise in mouse and human skeletal muscle (Sylow et al., 2013). Group I PAKs (PAK1‐3) are downstream targets of the Rho family GTPases Cdc42 and Rac1 (Manser, Leung, Salihuddin, Zhao, & Lim, 1994). Rac1 plays a key role in mediating GLUT4 translocation and consequently glucose uptake in response to muscle contraction and acute exercise in skeletal muscle (Sylow et al., 2013, 2016; Sylow, Møller, et al., 2017). Additionally, the contraction‐stimulated increase in PAK1/2 activity is blunted in skeletal muscles from muscle‐specific Rac1 knockout (KO) mice (Sylow et al., 2013), suggesting a role for PAK1 and/or PAK2 in regulating Rac1‐mediated effects during muscle contraction. Whereas PAK1 previously has been proposed to regulate insulin‐stimulated GLUT4 translocation in skeletal muscle (Tunduguru et al., 2014; Wang, Oh, Clapp, Chernoff, & Thurmond, 2011), we recently identified PAK2, but not PAK1, as a partial requirement for insulin‐stimulated glucose transport in mouse extensor digitorum longus (EDL) muscle (Moller et al., 2019). However, the significance of the increased activity of group I PAKs in the regulation of contraction‐stimulated muscle glucose transport is unknown. We hypothesized that PAK1 and PAK2 participate in the regulation of glucose transport in response to muscle contraction, due to their well‐described role as Rac1 effector proteins. Our results identify PAK2, but not PAK1, as a partial requirement for contraction‐stimulated glucose transport in mouse skeletal muscle.

## 2 MATERIALS AND METHODS

### 2.1 Animal experiments

All animal experiments complied with the European Convention for the protection of vertebrate animals used for experimental and other scientific purposes (No. 123, Strasbourg, France, 1985; EU Directive 2010/63/EU for animal experiments) and were approved by the Danish Animal Experimental Inspectorate. All mice were maintained on a 12:12‐hr light‐dark cycle and housed at 22°C (with allowed fluctuation of ±2°C) with nesting material. Female C57BL/6J mice (Taconic, Denmark) were used for the inhibitor incubation study. The mice received a standard rodent chow diet (Altromin no. 1324; Brogaarden) and water ad libitum. The mice were group housed.

### 2.2 Double PAK1^−/−^; PAK2^fl/fl^; MyoD^iCre/+^ mice

Double knockout mice with whole‐body knockout of PAK1 and conditional, muscle‐specific knockout of PAK2; PAK1^−/−^; PAK2^fl/fl^; MyoD^iCre/+^ were generated as previously described (Joseph et al., 2017). The mice were on a mixed C57BL/6/FVB background. PAK1^−/−^; PAK2^fl/fl^; MyoD^iCre/+^ were crossed with PAK1^+/−^; PAK2^fl/fl^; MyoD^+/+^ to generate littermate PAK1^−/−^; PAK2^fl/fl^; MyoD^iCre/+^ (referred to as 1/m2 dKO), PAK1^−/−^; PAK2^fl/fl^; MyoD^+/+^ (referred to as PAK1 KO), PAK1^+/−^; PAK2^fl/fl^; MyoD^iCre/+^ (referred to as PAK2 mKO), and PAK1^+/−^; PAK2^fl/fl^; MyoD^+/+^ (referred to as controls) used for experiments as previously described (Moller et al., 2019). Age‐matched littermate mice were used for experiments. At 26–35 weeks of age, female and male mice were used for the measurement of contraction‐stimulated glucose transport in isolated muscle. Male and female mice showed comparable phenotypes, thus data were pooled to increase statistical power and accuracy of the effect‐size estimation. Number of mice in each group: Control, *n = *6/4 (female/male); PAK1 KO, *n = *6/6; PAK2 mKO, *n = *6/7; 1/m2 dKO, *n = *6/7. Additional mice included for measurement of muscle mass: Control, *n = *0/0 (female/male); PAK1 KO, *n = *0/1; PAK2 mKO, *n = *3/0; 1/m2 dKO, *n = *2/2. The mice received standard rodent chow diet and water ad libitum. The mice were single‐caged 4–7 weeks prior to the isolation of muscles. The whole‐body metabolic characteristics for this cohort of mice, including insulin and glucose tolerance, have previously been described (Moller et al., 2019).

### 2.3 Mouse genotyping by PCR

An ear punch was digested overnight in 100 µl Viagen lysis buffer plus Proteinase K at 55°C followed by 45 min at 85°C. After spin at 1,000 × *g* for 5 min, the supernatant was diluted 10 times in TE (pH 8.0) with yellow color (50 pg/ml Quinoline Yellow). Five µl of this was used in a 25 µl real‐time quantitative PCR reaction containing Quantitect SYBR Green Master Mix (Qiagen), 200 nM of each primer (Table 1), and blue dye (5 pg/ml Xylene Cyanol). The reactions were furthermore spiked (100 times less than the samples) with a heterozygote sample as a positive PCR control. The samples, including no sample controls (TE), were amplified in an MX3005P real‐time PCR machine (95°C, 10 min → {95°C, 15 s → 58°C, 30 s → 63°C, 90 s} × 50 → melting curve 55°C → 95°C). The Ct values were used to access allele presence by comparison to the no DNA controls (spike values) such that the Ct value should be at least 2 Ct below the no sample controls to indicate the presence of the allele. Amplification efficiency in the individual reactions was estimated by the sigmoid method of Liu and Saint (2002) to ensure that the Ct's could be compared within primer sets. The genotype was later verified by immunoblotting on samples from muscle tissue.

**TABLE 1:** Primers sequences used for mouse genotyping

Gene target	Forward primer (5’‐3’)	Reverse primer (5’‐3’)
*Pak1* wild type	CCCCCGCAGCAAATAAAAAGA	CCCTGTGACAGCATCAAAACCA
*Pak1* floxed	CCCCCGCAGCAAATAAAAAGA	GGAAAAGCGCCTCCCCTACC
*Pak2* wild type	GAATGAAGCCCGAGTTCAAGTCCC	CTGCATCAATCTATTCTGACTATGACAGGT
*Pak2* floxed	TGCAGGTGCAGTGTGACAGAGA	TGAGCGGATCCACCTAATAACTTCGT
*MyoD* wild type	GCTCAGGAGGATGAGCAATGGA	ATAAGGGACACCCCCACCCCAAG
*MyoD* iCre	GGATCCGAATTCGAAGTTCCTATTCTCT	CCAAGGGCCTCGGAAACCTG

### 2.4 Incubation of isolated muscles

Soleus and EDL muscles were dissected from anaesthetized mice (6 mg pentobarbital sodium 100 g^−1^ body weight i.p.) and suspended at resting tension (4–5 mN) in incubations chambers (Multi Myograph System, Danish Myo Technology, Denmark) in Krebs‐Ringer‐Henseleit buffer with 2 mM pyruvate and 8 mM mannitol at 30°C, as described previously (Jørgensen et al., 2004). Additionally, the Krebs‐Ringer‐Henseleit buffer was supplemented with 0.1% BSA (v/v). Isolated muscles from female C57BL/6J mice were preincubated with 40 µM IPA‐3 (Sigma‐Aldrich) or a corresponding amount of DMSO (0.11%) for 45 min followed by 15 min of electrically stimulated contractions. Isolated muscles from PAK1 KO, PAK2 mKO, 1/m2 dKO, or littermate controls were preincubated 10–20 min followed by 15 min of electrically stimulated contractions. Contractions were induced by electrical stimulation every 15 s with 2‐s trains of 0.2 msec pulses delivered at 100 Hz (~35V) for 15 min. 2‐deoxyglucose (2DG) transport was measured together with 1 mM 2DG during the last 10 min of the contraction stimulation period using 0.60–0.75 µCi/ml [^3^H]‐2DG and 0.180–0.225 µCi/ml [^14^C]‐mannitol radioactive tracers (Perkin Elmer) essentially as described in (Jørgensen et al., 2004). Muscle‐specific [^3^H]‐2DG accumulation was measured in the lysate with [^14^C]‐mannitol as an extracellular marker and related to the specific activity of the incubation buffer.

### 2.5 Muscle processing

All muscles were homogenized 2 × 30 s at 30 Hz using a Tissuelyser II (Qiagen) in ice‐cold homogenization buffer (10% (v/v) Glycerol, 1% (v/v) NP‐40, 20 mM Na‐pyrophosphate, 150 mM NaCl, 50 mM HEPES (pH 7.5), 20 mM β‐glycerophosphate, 10 mM NaF, 2mM PMSF, 1 mM EDTA (pH 8.0), 1 mM EGTA (pH 8.0), 2 mM Na_3_VO_4_, 10 µg/ml Leupeptin, 10 µg/ml Aprotinin, and 3 mM Benzamidine). Homogenates were rotated end‐over‐end for 30 min at 4°C, and lysate (supernatant) generated by centrifugation (10,854–15,630 × g) for 15–20 min at 4°C .

### 2.6 Immunoblotting

Lysate protein concentration was determined using the bicinchoninic acid method using bovine serum albumin (BSA) standards and bicinchoninic acid assay reagents (Pierce). Immunoblotting samples were prepared in 6X sample buffer (340 mM Tris (pH 6.8), 225 mM DTT, 11% (w/v) SDS, 20% (v/v) Glycerol, and 0.05% (w/v) Bromphenol blue). Protein phosphorylation (p) and total protein expression were determined by standard immunoblotting technique loading equal amounts of protein. The polyvinylidene difluoride membrane (Immobilon Transfer Membrane; Millipore) was blocked in Tris‐Buffered Saline with added Tween20 (TBST) and 2% (w/v) skim milk or 3% BSA for 15 min at room temperature, followed by incubation overnight at 4°C with a primary antibody (Table 2). Next, the membrane was incubated with horseradish peroxidase‐conjugated secondary antibody (Jackson Immuno Research) for 1 hr at room temperature. Total ACC was detected without the use of antibodies. Instead, the membrane was incubated with horseradish peroxidase‐conjugated streptavidin (P0397; Dako; 1:3,000, 3% BSA) at 4°C overnight. Bands were visualized using Bio‐Rad ChemiDocTM MP Imaging System and enhanced chemiluminescence (ECL+; Amersham Biosciences). Actin protein expression or Coomassie Brilliant Blue staining was utilized as a control to assess total protein loading (Welinder & Ekblad, 2011) and for each sample set, a representative membrane from the immunoblotting is shown. Densitometric analysis was performed using Image LabTM Software, version 4.0 (Bio‐Rad; RRID: SCR_014210). For total protein expression, each data point was presented as the average of the protein expression in the left and right muscle from the same mouse.

**TABLE 2:** Antibodies used for immunoblotting

Antibody name	Antibody ID (RRID)	Manufacturer; catalog number;	Species raised in; monoclonal or polyclonal	Antibody dilution	Blocking buffer
pACC1/2 S79/212^a^	AB_330337	Cell Signaling Technology; 3,661	Rabbit; Polyclonal antibody	1:500	2% milk
Actin	AB_476693	Sigma‐Aldrich; A2066	Rabbit; Polyclonal antibody	1:10,000	2% milk
AMPKα2	AB_2169716	Santa Cruz Biotechnology; sc‐19131	Goat; Polyclonal antibody	1:1,000	2% milk
pAMPKα T172	AB_330330	Cell Signaling Technology; 2,531	Rabbit, Polyclonal antibody	1:1,000	2% milk
GLUT4	AB_2191454	Thermo Fisher Scientific; PA1−1065	Rabbit; Polyclonal antibody	1:1,000	2% milk
HKII	AB_2295219	Santa Cruz Biotechnology; Sc−130358	Mouse; Monoclonal antibody	1:1,000	2% milk
PAK1	AB_330222	Cell Signaling Technology; 2,602	Rabbit; Polyclonal antibody	1:500	2% milk
PAK2	AB_2283388	Cell Signaling Technology; 2,608	Rabbit; Polyclonal antibody	1:500	2% milk
TBC1D1	AB_2814949	Abcam; ab229504	Rabbit; Polyclonal	1 µg/µl	2% milk
pTBC1D1 S231^a^	AB_10807809	Millipore; 07‐2268	Rabbit; Polyclonal antibody	1:1,000	2% milk

^a^ Mouse nomenclature was used for pACC1/2 S79/212 (equivalent to human S80/221) and pTBC1D1 S231 (equivalent to human S237).

### 2.7 Statistical analyses

Data are presented as mean ± *SEM*. or when applicable, mean ± *SEM* with individual data points shown. Statistical tests varied according to the dataset being analyzed and the specific tests used are indicated in the figure legends. Datasets were normalized by square root, log10, or inverse transformation if not normally distributed or failed equal variance test. If the null hypothesis was rejected, Tukey's post hoc test was used to evaluate significant main effects of genotype and significant interactions in ANOVAs. *p* < .05 was considered statistically significant. *p* < .1 was considered a tendency. Except for mixed‐effects model analyses performed in GraphPad Prism, version 8.2.1. (GraphPad Software; RRID: SCR_002798), all statistical analyses were performed using Sigma Plot, version 13 (Systat Software Inc.; RRID: SCR_003210). Due to missing data points, differences between genotypes and the effect of electrically stimulated contractions were assessed with a mixed‐effects model analysis in Figure 2h+i.

## 3 RESULTS

### 3.1 Contraction‐stimulated glucose transport is partially inhibited by pharmacological inhibition of PAK1/2

**FIGURE 1:**
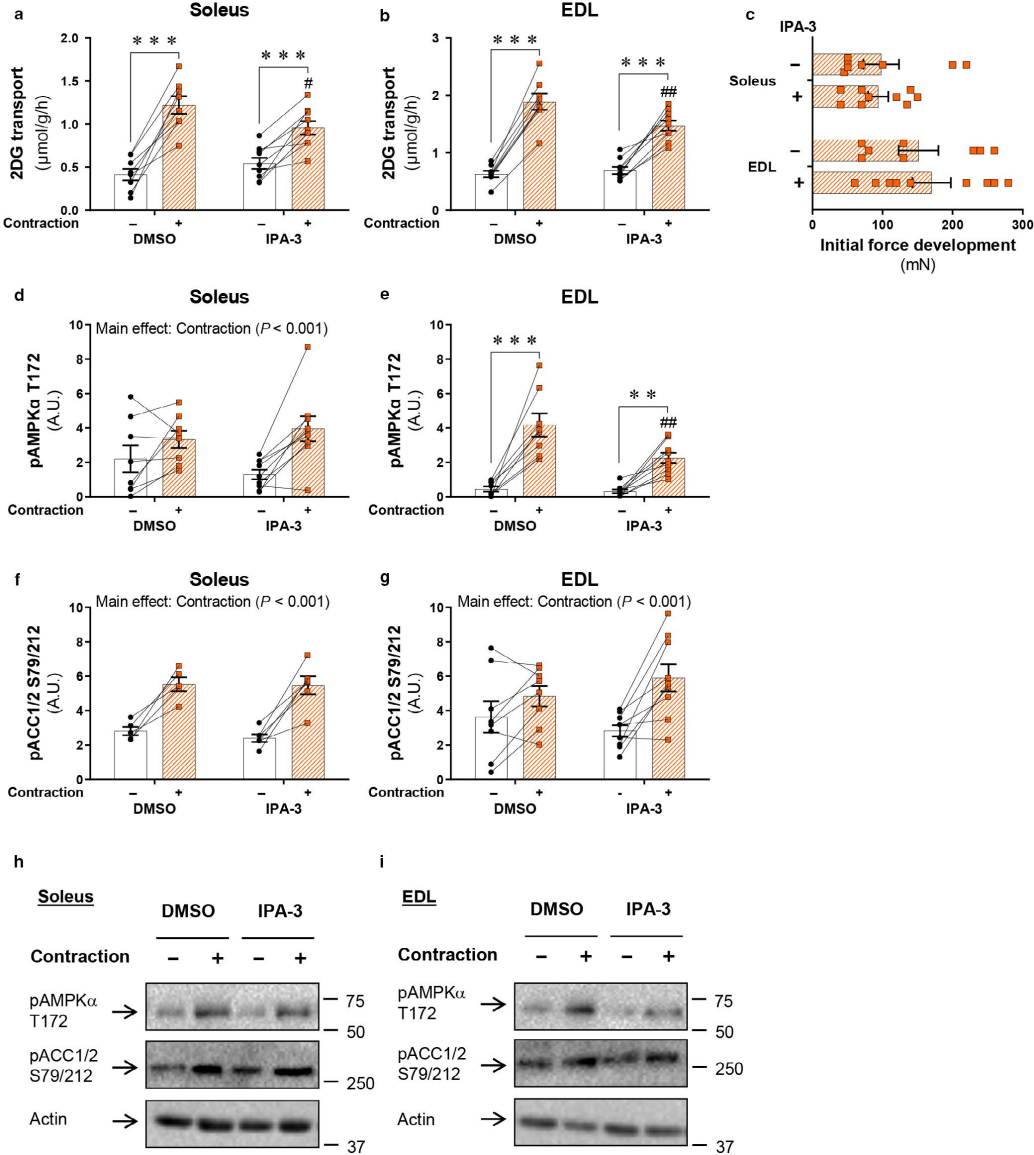
Contraction‐stimulated glucose transport is partially inhibited by pharmacological inhibition of PAK1/2. (a and b) Contraction‐stimulated (2 s/15 s, 100 Hz) 2‐deoxyglucose (2DG) transport in isolated soleus (a) and extensor digitorum longus (EDL; b) muscle ± 40 µM IPA‐3 or a corresponding amount of DMSO (0.11%). Isolated muscles were preincubated for 45 min followed by 15 min of electrically stimulated contractions with 2DG transport measured for the final 10 min of stimulation. Data were evaluated with a two‐way repeated‐measures (RM) ANOVA. (c) Initial force development during electrically stimulated contractions. Data were evaluated with a Student's *t* test. (d–g) Quantification of phosphorylated (p)AMPKα T172 and pACC1/2 S79/212 in contraction‐stimulated soleus (d and f) and EDL (e and g) muscles. Data were evaluated with a two‐way RM ANOVA. Some of the data points were excluded due to the quality of the immunoblot, and the number of determinations was *n* = 5/6 (DMSO/IPA‐3) for pACC1/2 S79/212 in soleus muscle. (h–i) Representative blots showing pAMPKα T172 and pACC1/2 S79/212 and actin protein expression as a loading control in soleus (h) and EDL (i) muscles. Main effects are indicated in the panels. Interactions in two‐way RM ANOVA were evaluated by Tukey's post hoc test: Contraction versus basal **/*** (*p* < .01/.001); IPA‐3 vs. DMSO #/## (*p* < .05/.01). Unless stated previously in the figure legend, the number of determinations in each group: Soleus, *n* = 8/9 (DMSO/IPA‐3); EDL, *n* = 8/9. Data are presented as mean ± *SEM* with individual data points shown. Paired data points are connected with a straight line. A.U., arbitrary units

To investigate the role of group I PAKs in the regulation of contraction‐stimulated glucose transport, we first analyzed 2‐deoxyglucose (2DG) transport in isolated soleus and EDL muscle in the presence or absence of a pharmacological group I PAK inhibitor, IPA‐3. Electrically stimulated contractions increased 2DG transport in DMSO‐treated soleus (2.9‐fold) and EDL (3.0‐fold) muscles (Figure 1a+b). IPA‐3 partly inhibited contraction‐stimulated 2DG transport in both soleus (−22%) and EDL (−22%; Figure 1a+b). The reduction in contraction‐stimulated 2DG transport upon IPA‐3 treatment was not associated with reduced initial force development in IPA‐3‐treated muscles (Figure 1c). While phosphorylated (p)AMPKα T172 was unaffected by IPA‐3 in soleus muscle (Figure 1d), contraction‐stimulated pAMPKα T172 was reduced (−46%) in IPA‐3‐treated EDL muscle (Figure 1e). However, AMPK’s downstream target pACC1/2 S79/212 was normally phosphorylated in response to electrically induced contractions in both muscles (Figure 1f+g), suggesting that the AMPK‐ACC signaling pathway was largely unaffected by IPA‐3 treatment. Altogether, these data suggest that pharmacological inhibition of group I PAKs partly reduces contraction‐stimulated glucose transport in mouse skeletal muscles.

### 3.2 Contraction‐stimulated glucose transport partially relies on PAK2, but not PAK1, in mouse EDL muscle

**FIGURE 2:**
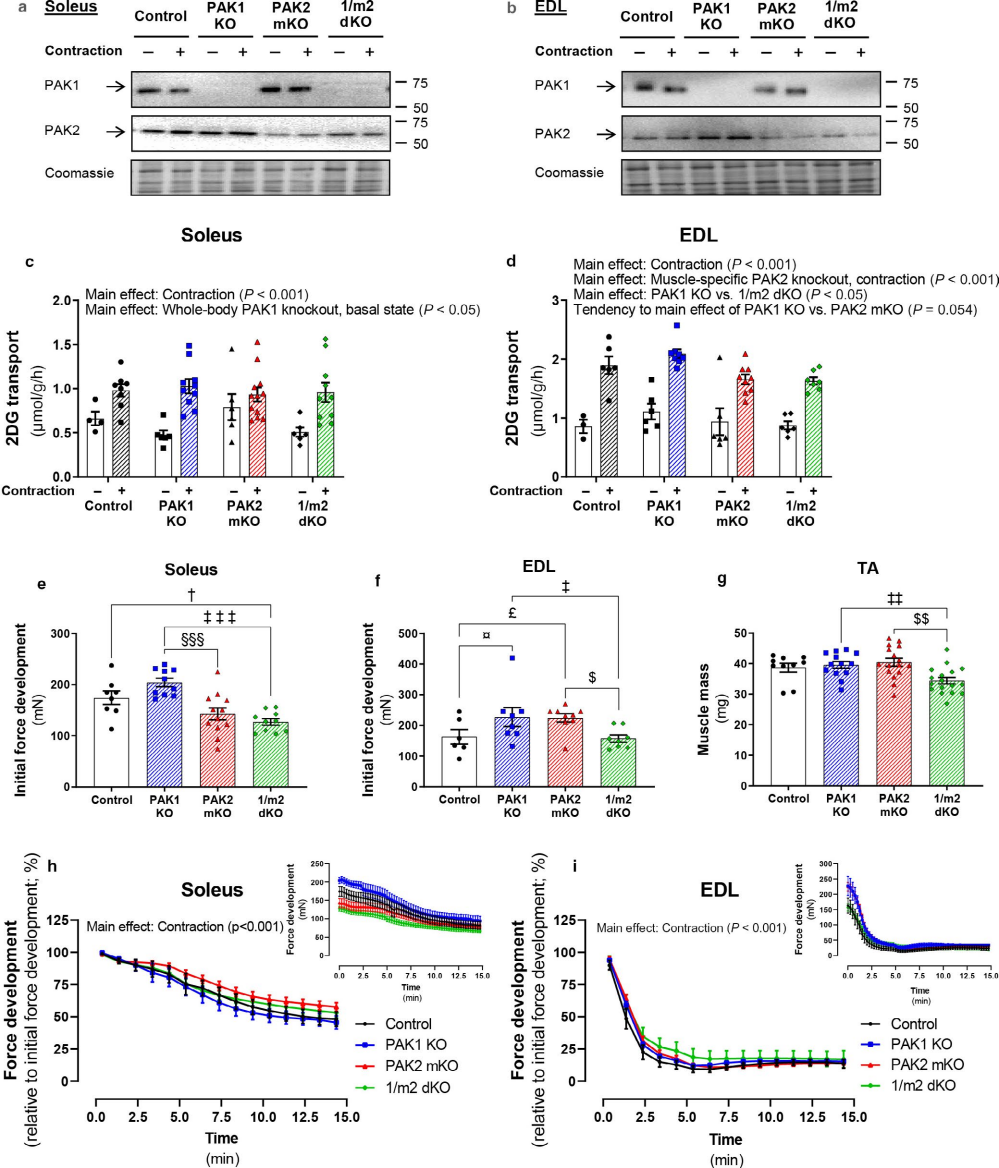
Contraction‐stimulated glucose transport partially requires PAK2, but not PAK1, in mouse EDL muscle. (a and b) Representative blots showing PAK1 and PAK2 protein expression in soleus (a) and extensor digitorum longus (EDL; b) muscles from whole‐body PAK1 knockout (KO), muscle‐specific PAK2 (m)KO, PAK1/2 double KO (1/m2 dKO) mice, or control littermates. Coomassie Brilliant Blue staining was utilized to assess total protein loading. Coomassie Brilliant Blue staining of a representative membrane from the immunoblotting of the sample set is shown. (c and d) Contraction‐stimulated (2 s/15 s, 100 Hz) 2‐deoxyglucose (2DG) transport in isolated soleus (c) and EDL (d) muscles from PAK1 KO, PAK2 mKO, 1/m2 dKO mice, or control littermates. Isolated muscles were preincubated for 10–20 min followed by 15 min of electrically stimulated contractions with 2DG transport measured for the final 10 min of stimulation. The number of determinations for soleus: Control, *n* = 4/8 (Basal/Contraction); PAK1 KO, *n* = 6/10; PAK2 mKO, *n* = 6/12; 1/m2 dKO, *n* = 6/10. The number of determinations for EDL: Control, *n* = 3/6 (Basal/Contraction); PAK1 KO, *n* = 6/8; PAK2 mKO, *n* = 6/9; 1/m2 dKO, *n* = 6/7. Data were evaluated with two two‐way ANOVAs to test the factors “PAK1” (PAK1^+/−^ vs. PAK^−/−^) and “PAK2” (PAK2^fl/fl^; MyoD^+/+^ vs. PAK2^fl/fl^; MyoD^iCre/+^) in the basal and contraction‐stimulated state, respectively, thereby assessing the relative contribution of PAK1 and PAK2. Differences between genotypes and the effect of electrically stimulated contractions were assessed with a two‐way ANOVA to test the factors “Genotype” (Control vs. PAK1 KO vs. PAK2 mKO vs. 1/m2 dKO) and “Stimuli” (Basal vs. Contraction). (e and f) Initial force development during electrically stimulated contractions in soleus (e) and EDL (f) muscles. The number of determinations in each group: Soleus, *n* = 8/10/12/10 (Control/PAK1 KO/PAK2 mKO/1/m2 dKO); EDL, *n* = 6/8/9/8. Data were evaluated with a two‐way ANOVA to test the factors “PAK1” and “PAK2,” thereby assessing the relative contribution of PAK1 and PAK2. Differences between genotypes were evaluated with a one‐way ANOVA. (g) Tibialis anterior (TA) muscle mass in PAK1 KO (*n* = 13), PAK2 mKO (*n* = 16), 1/m2 dKO mice (*n* = 17), or control littermates (*n* = 10). Data were evaluated with a two‐way ANOVA to test the factors “PAK1” and “PAK2,” thereby assessing the relative contribution of PAK1 and PAK2. Differences between genotypes were evaluated with a one‐way ANOVA. (h and i) Force development relative to initial force development in soleus (h) and EDL (i) muscles. Data points relative to initial force development are an average of the values at four consecutive time points. Original force development data inserted in the upper right corner. The number of determinations in each group: Soleus, *n* = 8/10/12/10 (Control/PAK1 KO/PAK2 mKO/1/m2 dKO); EDL, *n* = 6/8/9/8. Due to missing data points, differences between genotypes and the effect of electrically‐stimulated contractions were assessed with a mixed‐effects model analysis to test the factors “Genotype” and “Time point”. Main effects are indicated in the panels. Significant one‐way ANOVA and interactions in two‐way ANOVA (or mixed‐effects model) were evaluated by Tukey's post hoc test: Control versus PAK1 KO ¤ (*p* < .05); Control versus PAK2 mKO £ (*p* < .05); Control versus 1/m2 dKO † (*p* < .05); PAK1 KO versus PAK2 mKO §§§ (*p* < .001); PAK1 KO versus 1/m2 dKO ‡/‡‡/‡‡‡ (*p* < .05/.01/.001); PAK2 mKO versus 1/m2 dKO $/$$ (*p* < .05/.01). Data are presented as mean ± *SEM* with individual data points shown. Paired data points are connected with a straight line

IPA‐3 is a pharmacological inhibitor of group I PAKs (PAK1‐3) of which PAK1 and PAK2 are detectable in skeletal muscle (Arias‐Romero & Chernoff, 2008; Joseph et al., 2017; Tunduguru et al., 2014). To identify the relative role of PAK1 and PAK2 in the regulation of contraction‐stimulated glucose transport, we investigated contraction‐stimulated glucose transport in isolated soleus and EDL muscles from a cohort of whole‐body PAK1 KO, muscle‐specific PAK2 (m)KO, and double knockout mice with whole‐body knockout of PAK1 and muscle‐specific knockout of PAK2 (1/m2 dKO) compared to control littermates. PAK1 was not detectable at the protein level in muscles from mice with whole‐body knockout of PAK1 (i.e., PAK1 KO and 1/m2 dKO mice) (Figure 2a+b, Figure S1a and b). On the contrary, muscles lacking PAK2 (i.e., PAK2 mKO and 1/m2 dKO mice) displayed only a partial reduction in PAK2 protein expression (Figure 2a+b, Figure S1c and d). As the knockout of PAK2 is muscle specific, other cell types within skeletal muscle tissue contribute to the signal obtained in the PAK2 immunoblots. Moreover, the slightly upregulated PAK2 protein expression in EDL muscle lacking PAK1 (Figure 2b, Figure S1d) could be ascribed to a compensatory upregulation of PAK2 in muscle tissue (PAK1 KO mice) and nonmuscle tissue (PAK1 KO and 1/m2 dKO mice). The whole‐body metabolic characteristics of this cohort of mice have previously been described (Moller et al., 2019).

In soleus muscle, contraction‐stimulated glucose transport was unaffected by the lack of PAK1, PAK2, or both PAKs combined (Figure 2c). In contrast, in EDL lack of PAK2, either alone or in combination with PAK1 KO, partially reduced contraction‐stimulated glucose transport compared to PAK1 KO mice (PAK2 mKO: −21%; 1/m2 dKO: −22%) and control littermates (PAK2 mKO: −13%; 1/m2 dKO: −14%; Figure 2d). Lack of PAK2 decreased initial force development in soleus compared to PAK1 KO mice (PAK2 mKO: −30%; 1/m2 dKO: −38%) and control littermates (1/m2 dKO: −27%; Figure 2e). In EDL, lack of PAK1 (+40%) or PAK2 (+38%) alone increased initial force development, while combined knockout of PAK1 and PAK2 decreased initial force development compared to PAK1 KO mice (−31%) and PAK2 mKO mice (−30%; Figure 2f). Due to the technicalities in the measurement of 2DG transport, it was not possible to obtain accurate measures of the total soleus and EDL muscle mass. Thus, we are unable to normalize muscle tension to mass. However, previous investigations have reported atrophy in several distinct muscles, including soleus and EDL, from 1/m2 dKO mice (Joseph et al., 2017, 2019) and we also observed reduced muscle mass in tibialis anterior (TA) 1/m2 dKO muscles (−13%; Figure 2g). However, although the reduction in initial force development in 1/m2 dKO muscle potentially could be ascribed to muscle wasting, the decrease in force development over time was similar between all four genotypes in both soleus and EDL muscle (Figure 2h+i). Taken together, similar to insulin‐stimulated glucose uptake (Moller et al., 2019), PAK1 is dispensable for contraction‐stimulated glucose transport, while contraction‐stimulated glucose transport partially relies on PAK2 in glycolytic EDL muscle.

### 3.3 Canonical contraction signaling is largely unaffected by the lack of PAK1 and PAK2

**FIGURE 3:**
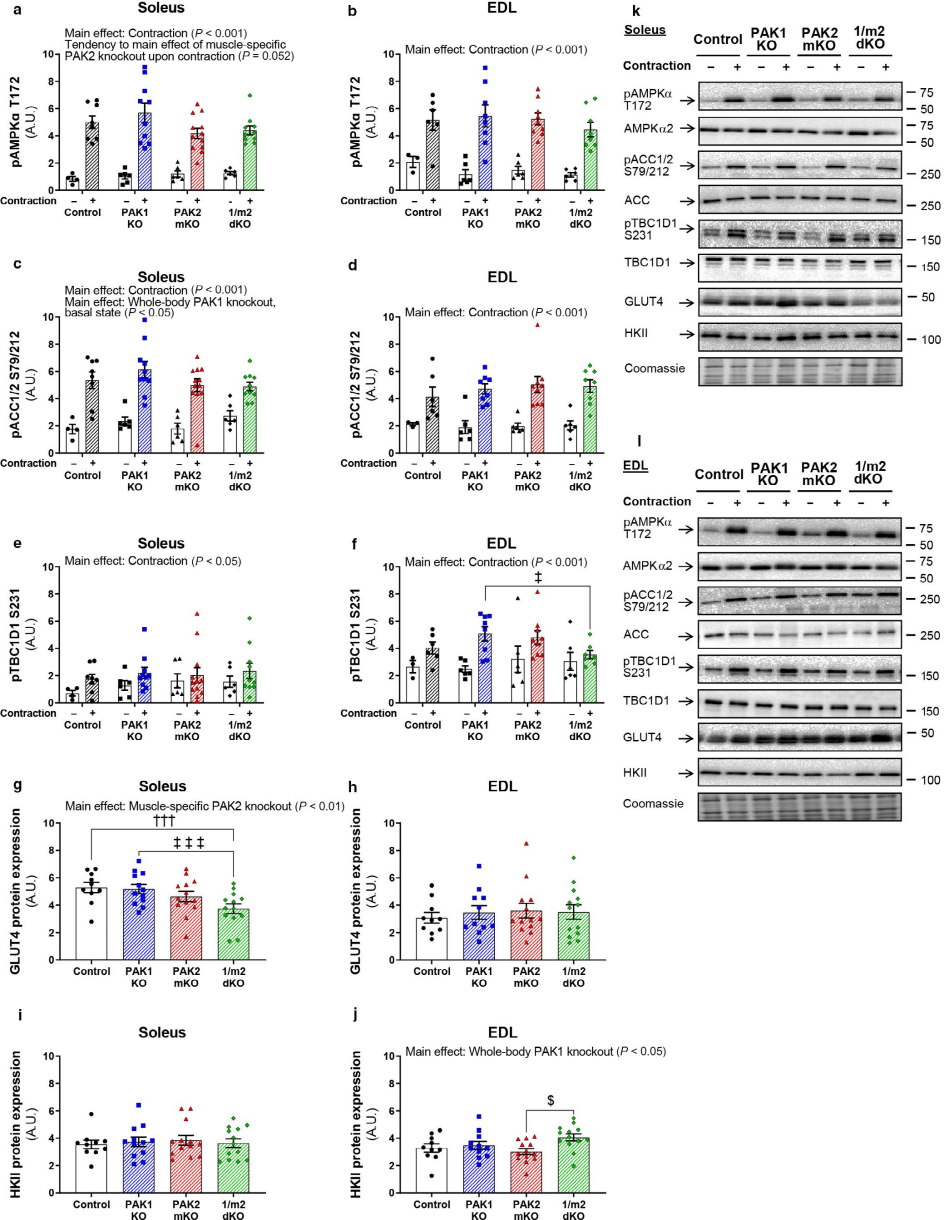
Canonical contraction signaling is largely unaffected by the lack of PAK1 and PAK2. (a–j) Quantification of phosphorylated (p)AMPKα T172, pACC1/2 S79/212, pTBC1D1 S231, and total GLUT4 and Hexokinase II (HKII) protein expression in response to electrically stimulated contractions (2 s/15 s, 100 Hz) in soleus (a, c, e, g, and i) and extensor digitorum longus (EDL; b, d, f, h, and j) muscles from whole‐body PAK1 knockout (KO), muscle‐specific PAK2 (m)KO, PAK1/2 double KO (1/m2 dKO) mice, or control littermates. Total protein expression is an average of the expression in the left and right muscle from the same mouse. Protein phosphorylation was evaluated with two two‐way ANOVAs to test the factors “PAK1” (PAK1^+/−^ vs. PAK^−/−^) and “PAK2” (PAK2^fl/fl^; MyoD^+/+^ vs. PAK2^fl/fl^; MyoD^iCre/+^) in basal and contraction‐stimulated samples, respectively, thereby assessing the relative contribution of PAK1 and PAK2. Differences between genotypes and the effect of electrically stimulated contractions stimulation were assessed with a two‐way ANOVA to test the factors “Genotype” (Control vs. PAK1 KO vs. PAK2 mKO vs. 1/m2 dKO) and “Stimuli” (Basal vs. Contraction). Total protein expression was evaluated with a two‐way ANOVA to test the factors “PAK1” and “PAK2” thereby assessing the relative contribution of PAK1 and PAK2. Differences between genotypes were evaluated with a one‐way ANOVA. (k and l) Representative blots showing pAMPKα T172, pACC1/2 S79/212, pTBC1D1 S231, and total AMPKα2, ACC, TBC1D1, GLUT4, and HKII protein expression in soleus (k) and EDL (l) muscles. Coomassie Brilliant Blue staining was utilized to assess total protein loading. Coomassie Brilliant Blue staining of a representative membrane from the immunoblotting of the sample set is shown. Main effects are indicated in the panels. Significant one‐way ANOVA and interactions in two‐way ANOVA were evaluated by Tukey's post hoc test: Control versus 1/m2 dKO ††† (*p* < .001); PAK1 KO versus 1/m2 dKO ‡/‡‡‡ (*p* < .05/.001); PAK2 mKO versus 1/m2 dKO $ (*p* < .05). For protein phosphorylation, the number of determinations for soleus: Control, *n = *4/8 (Basal/Contraction); PAK1 KO, *n* = 6/10; PAK2 mKO, *n = *6/12; 1/m2 dKO, *n = *6/10. The number of determinations for EDL: Control, *n* = 3/6 (Basal/Contraction); PAK1 KO, *n = *6/8; PAK2 mKO, *n* = 6/9; 1/m2 dKO, *n* = 6/8. For EDL, one data point from contraction‐stimulated pTBC1D1 S231 in 1/m2 dKO muscle is missing due to a lack of sample. For total protein expression, the number of determinations in each group: Soleus, *n* = 10/12/13/13 (Control/PAK1 KO/PAK2 mKO/1/m2 dKO); EDL, *n* = 10/11/13/13. Data are presented as mean ± *SEM* with individual data points shown. A.U., arbitrary units

Next, we investigated the effects of lack of PAK1 and/or PAK2 on contraction‐stimulated molecular signaling. Lack of PAK2 tended (*p* = .052) to reduce pAMPKα T172 in soleus (PAK2 mKO: −17%; 1/m2 dKO: −12%), but not EDL muscle (Figure 3a+b). However, pACC1/2 S79/212 was normally phosphorylated in response to contractions in both muscles (Figure 3c+d). Another contraction‐stimulated downstream target of AMPK, pTBC1D1 S231 was unaffected by lack of PAK1 and/or PAK2 in soleus muscle (Figure 3e), but was reduced (−39%) in EDL muscle from 1/m2 dKO mice compared to PAK1 KO mice (Figure 3f). Protein expression of AMPKα2, ACC, and TBC1D1 was unaffected by lack of PAK1 and/or PAK2 (representative blots in Figure 3k+l). We next analyzed the total protein content of proteins involved in glucose handling. Previously, in a slightly younger cohort (10–16 vs. 26–35 weeks of age), we reported that GLUT4 protein expression was normal in soleus but mildly reduced in EDL in PAK2 mKO mice compared to littermate controls (Moller et al., 2019). In contrast, GLUT4 protein expression was presently reduced in soleus 1/m2 dKO muscles compared to control muscles (−29%; Figure 3g). In EDL muscle, GLUT4 protein expression was unaffected by lack of PAK1 and/or PAK2 (Figure 3h). Some studies, but not all, report an age‐dependent decrease in GLUT4 expression in rodent and human skeletal muscle (Barnard et al., 1992; Cartee & Bohn, 1995; Cartee, Briggs‐Tung, & Kietzke, 1993; Cox, Cortright, Dohm, & Houmard, 1999; Dela et al., 1994; Ezaki, Higuchi, Nakatsuka, Kawanaka, & Itakura, 1992; Gaster, Poulsen, Handberg, Schrøder, & Beck‐Nielsen, 2000; Gulve, Rodnick, Henriksen, & Holloszy, 1993; Houmard et al., 1995; Kern, Dolan, Mazzeo, Wells, & Dohm, 1992; Lin et al., 1991; Sharma et al., 2010), potentially explaining the discrepancies in muscle GLUT4 protein expression between the two cohorts of mice. Protein expression of hexokinase II (HKII), a key enzyme converting glucose to glucose‐6‐phosphate after uptake, was unaffected in soleus muscle (Figure 3i), while higher (+34%) in 1/m2 dKO EDL muscle compared to PAK2 mKO muscle (Figure 3j). Taken together, the reduced contraction‐stimulated glucose transport in 1/m2 dKO EDL muscle was accompanied by impaired pTBC1D1 S237 phosphorylation (potentially decreasing glucose uptake) but also upregulation of HKII (potentially enhancing capacity for glucose uptake, although a previous study suggests that, in isolated muscles, HKII overexpression is not sufficient to increase neither basal nor insulin‐stimulated glucose transport (Hansen, Marshall, Chen, Holloszy, & Mueckler, 2000)). Thus, the mechanism/s by which genetic ablation of PAK2 reduces contraction‐stimulated glucose transport remain unclear.

## 4 DISCUSSION

The present study is, to our knowledge, the first to investigate the requirement of PAK1 and PAK2 in contraction‐stimulated glucose transport in mouse skeletal muscle. By undertaking a systematic investigation, including pharmacological as well as genetic interventions, we show that contraction‐stimulated glucose transport in isolated skeletal muscle partially requires PAK2, but not PAK1, in glycolytic EDL muscle.

In the current study, IPA‐3 attenuated the increase in muscle glucose transport in response to the electrically stimulated contractions in both soleus and EDL muscle, whereas genetically targeted knockout revealed an effect of PAK2 in glycolytic EDL only. It is not unusual that pharmacological inhibition and genetically targeted mutations produce different phenotypes (Knight & Shokat, 2007). This likely means that either the effect of the IPA‐3 on glucose transport in soleus is unspecific or, alternatively, that the absent effect of genetic ablation of PAK1 and/or PAK2 in soleus is due to compensation by other mechanisms. It is important to stress that any possible compensatory mechanisms cannot be via redundancy with PAK3, as even in 1/m2 dKO muscle, PAK3 cannot be detected at the protein level (Joseph et al., 2017). In contrast, a kinase screen of 214 full‐length human kinases revealed that 10 µM IPA‐3 significantly inhibited nine kinases (Deacon et al., 2008). Among these kinases, Akt2 and Glycogen Synthase Kinase (GSK)‐3α/β were identified. GSK‐3 is indirectly involved in glucose uptake via glycogen synthase regulation and glycogen deposition (Embi, Rylatt, & Cohen, 1980), whereas Akt2 is an established regulator of insulin‐stimulated glucose uptake in skeletal muscle (Cho et al., 2001; Garofalo et al., 2003; McCurdy & Cartee, 2005). However, despite the reported unspecific inhibition of Akt2 (Deacon et al., 2008), we and others have reported that insulin‐stimulated Akt phosphorylation is not significantly affected by IPA‐3 in muscle cells (Tunduguru et al., 2014) and mature mouse skeletal muscle (Moller et al., 2019). In contrast, due to its chemical structure, IPA‐3 has been proposed likely to alter the redox potential of cells because of the continuous reduction of IPA‐3 (Rudolph, Crawford, Hoeflich, & Chernoff, 2013), potentially causing group I PAK‐independent effects of IPA‐3. Interestingly, a recent analysis of the crosstalk between oxidation and protein phosphorylation in adipocytes suggested that oxidation of key regulatory kinases, including AMPK, influences the fidelity of the kinase (Su et al., 2019), while, in cardiomyocytes, the activity of AMPK has been suggested to be negatively regulated by oxidation (Shao et al., 2014). Thus, changed redox status due to the continuous reduction of IPA‐3 could be a possible explanation for the decrease in pAMPKα T172 phosphorylation observed in IPA‐3‐treated EDL muscle.

The limited role of group I PAKs in contraction‐induced glucose transport is in accordance with our recent finding that group I PAKs were largely dispensable for insulin‐stimulated glucose transport in isolated mouse skeletal muscle with only a modest reduction in EDL muscles lacking PAK2 (Moller et al., 2019). Thus, group I PAKs are not major essential components in the regulation of muscle glucose transport. Based on recent emerging evidence, the role for group I PAKs in skeletal muscle seems instead to be related to myogenesis and muscle mass regulation (Joseph et al., 2017, 2019). Additionally, in embryonic day 18.5 diaphragm, combined genetic ablation of PAK1 and PAK2 was associated with reduced acetylcholine receptor clustering at the neuromuscular junction, (Joseph et al., 2017) suggesting defects in the neuromuscular synapses.

This relatively modest requirement of group I PAKs in contraction‐induced muscle glucose transport is in contrast to the marked glucoregulatory role of Rac1 (Sylow et al., 2013, 2016; Sylow, Møller, et al., 2017), the upstream regulator of group I PAKs. Rac1 is an essential component in the activation of the reactive oxygen‐producing NADPH oxidase (NOX)‐2 complex (Abo et al., 1991; Bedard & Krause, 2007). Recently, it was reported that NOX2 is required for exercise‐stimulated glucose uptake (Henríquez‐Olguin et al., 2019). Moreover, it was shown that exercise‐induced NOX2 activation was completely abrogated in TA from muscle‐specific Rac1 KO mice (Henríquez‐Olguin et al., 2019), suggesting that Rac1 mainly regulates muscle glucose uptake through activation of NOX2 in response to exercise. Alternatively, the Ral family GTPase, RalA could signal downstream of Rac1. Overexpression of a constitutively activated Rac1 mutant activated RalA in L6 myotubes (Nozaki, Ueda, Takenaka, Kataoka, & Satoh, 2012). Additionally, GLUT4 translocation induced by a constitutively active Rac1 mutant was attenuated in L6‐GLUT4myc myoblasts upon RalA knockdown (Nozaki et al., 2012). The RalA GTPase‐activating protein GARNL1 is phosphorylated in response to in situ contraction of mouse skeletal muscle (Chen et al., 2014), but so far no linkage between Rac1 and RalA has been reported in relation to contraction‐stimulated glucose transport.

In conclusion, contraction‐stimulated glucose transport in isolated mouse skeletal muscle partially requires PAK2, but not PAK1, in glycolytic EDL muscle. Together with our previous study showing that insulin‐stimulated glucose transport also partially requires PAK2, but not PAK1 (Moller et al., 2019), this suggests that group I PAKs play at most a minor role in the regulation skeletal muscle glucose transport.

## CONFLICT OF INTEREST

No potential conflicts of interest relevant to this article were reported.

## AUTHOR CONTRIBUTIONS

LLVM: Conceptualization; Methodology; Formal analysis; Investigation; Writing – Original Draft; Writing – Review & Editing; Visualization; Project administration; Funding acquisition. ILN: Investigation; Writing – Review & Editing. JRK: Investigation; Writing – Review & Editing; Funding acquisition. NRA: Investigation; Writing – Review & Editing. TEJ: Investigation; Writing – Review & Editing; Funding acquisition. LS: Conceptualization; Methodology; Investigation; Writing – Original Draft; Writing – Review & Editing; Supervision; Project administration; Funding acquisition. EAR: Conceptualization; Methodology; Writing – Original Draft; Writing – Review & Editing; Supervision; Project administration; Funding acquisition. EAR is the guarantor of this work and takes responsibility for the integrity of the data and the accuracy of the data analysis.

## Supporting information



Figure S1

Caption

## Data Availability

The datasets generated and analyzed during the current study are available from the corresponding author upon reasonable request. No novel applicable resources were generated or analyzed during the current study.

## References

[phy214460-bib-0001] Abo, A. , Pick, E. , Hall, A. , Totty, N. , Teahan, C. G. , & Segal, A. W. (1991). Activation of the NADPH oxidase involves the small GTP‐binding protein p21rac1. Nature, 353(6345), 668–670. 10.1038/353668a0 1922386

[phy214460-bib-0002] Arias‐Romero, L. E. , & Chernoff, J. (2008). A tale of two Paks. Biology of the Cell, 100(2), 97–108. 10.1042/BC20070109 18199048

[phy214460-bib-0003] Barnard, R. J. , Lawani, L. O. , Martin, D. A. , Youngren, J. F. , Singh, R. , & Scheck, S. H. (1992). Effects of maturation and aging on the skeletal muscle glucose transport system. American Journal of Physiology‐Endocrinology and Metabolism, 262(5), E619–E626. 10.1152/ajpendo.1992.262.5.E619 1590372

[phy214460-bib-0004] Bedard, K. , & Krause, K.‐H. (2007). The NOX family of ROS‐generating NADPH oxidases: Physiology and pathophysiology. Physiological Reviews, 87(1), 245–313. 10.1152/physrev.00044.2005 17237347

[phy214460-bib-0005] Cartee, G. D. , & Bohn, E. E. (1995). Growth hormone reduces glucose transport but not GLUT1‐1 or GLUT‐4 in adult and old rats. American Journal of Physiology‐Endocrinology and Metabolism, 268(5), E902–E909. 10.1152/ajpendo.1995.268.5.e902 7762644

[phy214460-bib-0006] Cartee, G. D. , Briggs‐Tung, C. , & Kietzke, E. W. (1993). Persistent effects of exercise on skeletal muscle glucose transport across the life‐span of rats. Journal of Applied Physiology, 75(2), 972–978. 10.1152/jappl.1993.75.2.972 8226503

[phy214460-bib-0007] Chen, Q. , Quan, C. , Xie, B. , Chen, L. , Zhou, S. , Toth, R. , … Chen, S. . (2014). GARNL1, a major RalGAP α subunit in skeletal muscle, regulates insulin‐stimulated RalA activation and GLUT4 trafficking via interaction with 14‐3‐3 proteins. Cellular Signalling, 26(8), 1636–1648. 10.1016/j.cellsig.2014.04.012 24768767

[phy214460-bib-0008] Cho, H. , Mu, J. , Kim, J. K. , Thorvaldsen, J. L. , Chu, Q. , Crenshaw, E. B. , … Birnbaum, M. J. (2001). Insulin resistance and a diabetes mellitus‐like syndrome in mice lacking the protein kinase Akt2 (PKB beta). Science, 292(5522), 1728–1731. 10.1126/science.292.5522.1728 11387480

[phy214460-bib-0009] Cox, J. H. , Cortright, R. N. , Dohm, G. L. , & Houmard, J. A. (1999). Effect of aging on response to exercise training in humans: Skeletal muscle GLUT‐4 and insulin sensitivity. Journal of Applied Physiology, 86(6), 2019–2025. 10.1152/jappl.1999.86.6.2019 10368369

[phy214460-bib-0010] Deacon, S. W. , Beeser, A. , Fukui, J. A. , Rennefahrt, U. E. E. , Myers, C. , Chernoff, J. , & Peterson, J. R. . (2008). An Isoform‐selective, small‐molecule inhibitor targets the autoregulatory mechanism of p21‐activated kinase. Chemistry & Biology, 15(4), 322–331. 10.1016/j.chembiol.2008.03.005 18420139 PMC4353635

[phy214460-bib-0011] Dela, F. , Ploug, T. , Handberg, A. , Petersen, L. N. , Larsen, J. J. , Mikines, K. J. , & Galbo, H. . (1994). Physical training increases muscle GLUT4 protein and mRNA in patients with NIDDM. Diabetes, 43(7), 862–865. 10.2337/diab.43.7.862 8013748

[phy214460-bib-0012] Douen, A. G. , Ramlal, T. , Rastogi, S. , Bilan, P. J. , Cartee, G. D. , Vranic, M. , … Klip, A. (1990). Exercise induces recruitment of the ‘insulin‐responsive glucose transporter’. Evidence for distinct intracellular insulin‐ and exercise‐recruitable transporter pools in skeletal muscle. Journal of Biological Chemistry, 265(23), 13427–13430. 2199436

[phy214460-bib-0013] Embi, N. , Rylatt, D. B. , & Cohen, P. (1980). Glycogen synthase kinase‐3 from rabbit skeletal muscle. Separation from cyclic‐AMP‐dependent protein kinase and phosphorylase kinase. European Journal of Biochemistry, 107(2), 519–527. 10.1111/j.1432-1033.1980.tb06059.x 6249596

[phy214460-bib-0014] Ezaki, O. , Higuchi, M. , Nakatsuka, H. , Kawanaka, K. , & Itakura, H. (1992). Exercise training increases glucose transporter content in skeletal muscles more efficiently from aged obese rats than young lean rats. Diabetes, 41(8), 920–926. 10.2337/diab.41.8.920 1628766

[phy214460-bib-0015] Garofalo, R. S. , Orena, S. J. , Rafidi, K. , Torchia, A. J. , Stock, J. L. , Hildebrandt, A. L. , … Coleman, K. G. . (2003). Severe diabetes, age‐dependent loss of adipose tissue, and mild growth deficiency in mice lacking Akt2/PKBβ. Journal of Clinical Investigation, 112(2), 197–208. 10.1172/JCI16885 12843127 PMC164287

[phy214460-bib-0016] Gaster, M. , Poulsen, P. , Handberg, A. , Schrøder, H. D. , & Beck‐Nielsen, H. (2000). Direct evidence of fiber type‐dependent GLUT‐4 expression in human skeletal muscle. American Journal of Physiology‐Endocrinology and Metabolism, 278(5), E910–E916. 10.1152/ajpendo.2000.278.5.E910 10780948

[phy214460-bib-0017] Gulve, E. A. , Rodnick, K. J. , Henriksen, E. J. , & Holloszy, J. O. (1993). Effects of wheel running on glucose transporter (GLUT4) concentration in skeletal muscle of young adult and old rats. Mechanisms of Ageing and Development, 67(1–2), 187–200. 10.1016/0047-6374(93)90122-8 8469030

[phy214460-bib-0018] Hansen, P. A. , Gulve, E. A. , & Holloszy, J. O. (1994). Suitability of 2‐deoxyglucose for in vitro measurement of glucose transport activity in skeletal muscle. Journal of Applied Physiology, 76(2), 979–985. 10.1152/jappl.1994.76.2.979 8175614

[phy214460-bib-0019] Hansen, P. A. , Marshall, B. A. , Chen, M. , Holloszy, J. O. , & Mueckler, M. (2000). Transgenic overexpression of hexokinase ii in skeletal muscle does not increase glucose disposal in wild‐type or Glut1‐overexpressing mice. Journal of Biological Chemistry, 275(29), 22381–22386. 10.1074/jbc.M001946200 10764781

[phy214460-bib-0020] Henríquez‐Olguin, C. , Knudsen, J. R. , Raun, S. H. , Li, Z. , Dalbram, E. , Treebak, J. T. , … Jensen, T. E. (2019). Cytosolic ROS production by NADPH oxidase 2 regulates muscle glucose uptake during exercise. Nature Communications, 10(1), 10.1038/s41467-019-12523-9 PMC6789013 31604916

[phy214460-bib-0021] Houmard, J. A. , Weidner, M. D. , Dolan, P. L. , Leggett‐Frazier, N. , Gavigan, K. E. , Hickey, M. S. , … Dohm, G. L. . (1995). Skeletal muscle GLUT4 protein concentration and aging in humans. Diabetes, 44(5), 555–560. 10.2337/diab.44.5.555 7729615

[phy214460-bib-0022] Hu, F. , Li, N. , Li, Z. , Zhang, C. , Yue, Y. , Liu, Q. , … Niu, W. . (2018). Electrical pulse stimulation induces GLUT4 translocation in a Rac‐Akt‐dependent manner in C2C12 myotubes. FEBS Letters, 592(4), 644–654. 10.1002/1873-3468.12982 29355935

[phy214460-bib-0053] Jørgensen S. B. , Viollet B. , Andreelli F. , Frøsig C. , Birk J. B. , Schjerling P. , Wojtaszewski J. F. P. (2004). Knockout of the α2but Not α15′‐AMP‐activated Protein Kinase Isoform Abolishes 5‐Aminoimidazole‐4‐carboxamide‐1‐β‐4‐ribofuranosidebut Not Contraction‐induced Glucose Uptake in Skeletal Muscle. Journal of Biological Chemistry, 279(2), 1070–1079. 10.1074/jbc.m306205200 14573616

[phy214460-bib-0023] Joseph, G. A. , Hung, M. , Goel, A. J. , Hong, M. , Rieder, M. K. , Beckmann, N. D. , … Aldana‐Hernandez, P. (2019). Late‐onset megaconial myopathy in mice lacking group I Paks. Skeletal Muscle, 9(1), 5 10.1186/s13395-019-0191-4 30791960 PMC6383276

[phy214460-bib-0024] Joseph, G. A. , Lu, M. , Radu, M. , Lee, J. K. , Burden, S. J. , Chernoff, J. , & Krauss, R. S. . (2017). Group I Paks promote skeletal myoblast differentiation in vivo and in vitro. Molecular and Cellular Biology, 37(4), e00222‐16 10.1128/MCB.00222-16 PMC5288579 27920252

[phy214460-bib-0025] Kennedy, J. W. , Hirshman, M. F. , Gervino, E. V. , Ocel, J. V. , Forse, R. A. , Hoenig, S. J. , … Horton, E. S. . (1999). Acute exercise induces GLUT4 translocation in skeletal muscle of normal human subjects and subjects with type 2 diabetes. Diabetes, 48(5), 1192–1197. 10.2337/diabetes.48.5.1192 10331428

[phy214460-bib-0026] Kern, M. , Dolan, P. L. , Mazzeo, R. S. , Wells, J. A. , & Dohm, G. L. (1992). Effect of aging and exercise on GLUT‐4 glucose transporters in muscle. American Journal of Physiology‐Endocrinology and Metabolism, 263(2), E362–E367. 10.1152/ajpendo.1992.263.2.E362 1514619

[phy214460-bib-0028] Knight, Z. A. , & Shokat, K. M. (2007). Chemical genetics: Where genetics and pharmacology meet. Cell, 128(3), 425–430. 10.1016/j.cell.2007.01.021 17289560

[phy214460-bib-0029] Lin, J. L. , Asano, T. , Shibasaki, Y. , Tsukuda, K. , Katagiri, H. , Ishihara, H. , … Oka, Y. (1991). Altered expression of glucose transporter isoforms with aging in rats? Selective decrease in GluT4 in the fat tissue and skeletal muscle. Diabetologia, 34(7), 477–482. 10.1007/BF00403283 1916052

[phy214460-bib-0030] Liu, W. , & Saint, D. A. (2002). Validation of a quantitative method for real time PCR kinetics. Biochemical and Biophysical Research Communications, 294(2), 347–353. 10.1016/S0006-291X(02)00478-3 12051718

[phy214460-bib-0031] Lund, S. , Holman, G. D. , Schmitz, O. , & Pedersen, O. (1995). Contraction stimulates translocation of glucose transporter GLUT4 in skeletal muscle through a mechanism distinct from that of insulin. Proceedings of the National Academy of Sciences, 92(13), 5817–5821. 10.1073/pnas.92.13.5817 PMC41592 7597034

[phy214460-bib-0032] Manser, E. , Leung, T. , Salihuddin, H. , Zhao, Z. , & Lim, L. (1994). A brain serine/threonine protein kinase activated by Cdc42 and Rac1. Nature, 367(6458), 40–46. 10.1038/367040a0 8107774

[phy214460-bib-0033] Martin, I. K. , Katz, A. , & Wahren, J. (1995). Splanchnic and muscle metabolism during exercise in NIDDM patients. American Journal of Physiology‐Endocrinology and Metabolism, 269(3), E583–E590. 10.1152/ajpendo.1995.269.3.E583 7573437

[phy214460-bib-0034] McCurdy, C. E. , & Cartee, G. D. (2005). Akt2 is essential for the full effect of calorie restriction on insulin‐stimulated glucose uptake in skeletal muscle. Diabetes, 54(5), 1349–1356. 10.2337/diabetes.54.5.1349 15855319

[phy214460-bib-0035] Mikines, K. J. , Sonne, B. , Farrell, P. A. , Tronier, B. , & Galbo, H. (1988). Effect of physical exercise on sensitivity and responsiveness to insulin in humans. American Journal of Physiology‐Endocrinology and Metabolism, 254(3), E248–E259. 10.1152/ajpendo.1988.254.3.E248 3126668

[phy214460-bib-0036] Moller, L. L. , Jaurji, M. , Kjobsted, R. , Joseph, G. A. , Madsen, A. B. , Knudsen, J. R. , … Krauss, R. S. (2019). Insulin‐stimulated glucose uptake partly relies on p21‐activated kinase (PAK)‐2, but not PAK1, in mouse skeletal muscle. bioRxiv, 543736 10.1101/543736 PMC7771197 32844438

[phy214460-bib-0037] Nozaki, S. , Ueda, S. , Takenaka, N. , Kataoka, T. , & Satoh, T. (2012). Role of RalA downstream of Rac1 in insulin‐dependent glucose uptake in muscle cells. Cellular Signalling, 24(11), 2111–2117. 10.1016/j.cellsig.2012.07.013 22820503

[phy214460-bib-0038] Ploug, T. , Galbo, H. , & Richter, E. A. (1984). Increased muscle glucose uptake during contractions: No need for insulin. American Journal of Physiology‐Endocrinology and Metabolism, 247(6), E726–E731. 10.1152/ajpendo.1984.247.6.E726 6391198

[phy214460-bib-0039] Ploug, T. , van Deurs, B. , Ai, H. , Cushman, S. W. , & Ralston, E. (1998). Analysis of GLUT4 distribution in whole skeletal muscle fibers: Identification of distinct storage compartments that are recruited by insulin and muscle contractions. Journal of Cell Biology, 142(6), 1429–1446. 10.1083/jcb.142.6.1429 9744875 PMC2141761

[phy214460-bib-0040] Richter, E. A. , Garetto, L. P. , Goodman, M. N. , & Ruderman, N. B. (1982). Muscle glucose metabolism following exercise in the rat: Increased sensitivity to insulin. Journal of Clinical Investigation, 69(4), 785–793. 10.1172/JCI110517 6804492 PMC370132

[phy214460-bib-0041] Richter, E. A. , & Hargreaves, M. (2013). Exercise, GLUT4, and skeletal muscle glucose uptake. Physiological Reviews, 93(3), 993–1017. 10.1152/physrev.00038.2012 23899560

[phy214460-bib-0042] Richter, E. A. , Mikines, K. J. , Galbo, H. , & Kiens, B. (1989). Effect of exercise on insulin action in human skeletal muscle. Journal of Applied Physiology, 66(2), 876–885. 10.1152/jappl.1989.66.2.876 2496078

[phy214460-bib-0043] Rudolph, J. , Crawford, J. J. , Hoeflich, K. P. , & Chernoff, J. (2013). P21‐activated kinase inhibitors In The Enzymes (Vol. 34, pp. 157–180). Cambridge, MA: Academic Press 10.1016/B978-0-12-420146-0.00007-X 25034104

[phy214460-bib-0044] Shao, D. , Oka, S.‐I. , Liu, T. , Zhai, P. , Ago, T. , Sciarretta, S. , … Sadoshima, J. . (2014). A redox‐dependent mechanism for regulation of AMPK activation by thioredoxin1 during energy starvation. Cell Metabolism, 19(2), 232–245. 10.1016/j.cmet.2013.12.013 24506865 PMC3937768

[phy214460-bib-0045] Sharma, N. , Arias, E. B. , Saian, M. P. , MacKrell, J. G. , Bhat, A. D. , Farese, R. V. , & Cartee, G. D. . (2010). Insulin resistance for glucose uptake and Akt2 phosphorylation in the soleus, but not epitrochlearis, muscles of old vs. adult rats. Journal of Applied Physiology, 108(6), 1631–1640. 10.1152/japplphysiol.01412.2009 20339009 PMC2886681

[phy214460-bib-0046] Su, Z. , Burchfield, J. G. , Yang, P. , Humphrey, S. J. , Yang, G. , Francis, D. , … Astore, M. A. (2019). Global redox proteome and phosphoproteome analysis reveals redox switch in Akt. Nature Communications, 10(1), 1–8. 10.1038/s41467-019-13114-4 PMC6889415 31792197

[phy214460-bib-0047] Sylow, L. , Jensen, T. E. , Kleinert, M. , Mouatt, J. R. , Maarbjerg, S. J. , Jeppesen, J. , … Schjerling, P. (2013). Rac1 is a novel regulator of contraction‐stimulated glucose uptake in skeletal muscle. Diabetes, 62(4), 1139–1151. 10.2337/db12-0491 23274900 PMC3609592

[phy214460-bib-0048] Sylow, L. , Kleinert, M. , Richter, E. A. , & Jensen, T. E. (2017). Exercise‐stimulated glucose uptake — regulation and implications for glycaemic control. Nature Reviews Endocrinology, 13(3), 133–148. 10.1038/nrendo.2016.162 27739515

[phy214460-bib-0049] Sylow, L. , Møller, L. L. , Kleinert, M. , D’Hulst, G. , De Groote, E. , Schjerling, P. , … Richter, E. A. (2017). Rac1 and AMPK account for the majority of muscle glucose uptake stimulated by ex vivo contraction but not in vivo exercise. Diabetes, 66(6), 1548–1559. 10.2337/db16-1138 28389470

[phy214460-bib-0050] Sylow, L. , Nielsen, I. L. , Kleinert, M. , Møller, L. L. , Ploug, T. , Schjerling, P. , … Richter, E. A. (2016). Rac1 governs exercise‐stimulated glucose uptake in skeletal muscle through regulation of GLUT4 translocation in mice. The Journal of Physiology, 594(17), 4997–5008. 10.1113/JP272039 27061726 PMC5009787

[phy214460-bib-0051] Sylow, L. , & Richter, E. A. (2019). Current advances in our understanding of exercise as medicine in metabolic disease. Current Opinion in Physiology, 12, 12–19. 10.1016/j.cophys.2019.04.008

[phy214460-bib-0052] Tunduguru, R. , Chiu, T. T. , Ramalingam, L. , Elmendorf, J. S. , Klip, A. , & Thurmond, D. C. (2014). Signaling of the p21‐activated kinase (PAK1) coordinates insulin‐stimulated actin remodeling and glucose uptake in skeletal muscle cells. Biochemical Pharmacology, 92(2), 380–388. 10.1016/j.bcp.2014.08.033 25199455 PMC4418524

[phy214460-bib-0054] Wallberg‐Henriksson, H. , & Holloszy, J. O. (1985). Activation of glucose transport in diabetic muscle: Responses to contraction and insulin. American Journal of Physiology‐Cell Physiology, 249(3), C233–C237. 10.1152/ajpcell.1985.249.3.C233 3898862

[phy214460-bib-0055] Wang, Z. , Oh, E. , Clapp, D. W. , Chernoff, J. , & Thurmond, D. C. (2011). Inhibition or ablation of p21‐activated kinase (PAK1) disrupts glucose homeostatic mechanisms in vivo. Journal of Biological Chemistry, 286(48), 41359–41367. 21969371 10.1074/jbc.M111.291500 PMC3308848

[phy214460-bib-0056] Welinder, C. , & Ekblad, L. (2011). Coomassie staining as loading control in western blot analysis. Journal of Proteome Research, 10(3), 1416–1419. 10.1021/pr1011476 21186791

[phy214460-bib-0057] Wojtaszewski, J. F. , Hansen, B. F. , Kiens, B. , & Richter, E. A. (1997). Insulin signaling in human skeletal muscle: Time course and effect of exercise. Diabetes, 46(11), 1775–1781. 10.2337/diab.46.11.1775 9356025

[phy214460-bib-0058] Yue, Y. , Zhang, C. , Zhang, X. , Zhang, S. , Liu, Q. , Hu, F. , … Chen, L. (2019). An AMPK/Axin1‐Rac1 signaling pathway mediates contraction‐regulated glucose uptake in skeletal muscle cells. American Journal of Physiology‐Endocrinology and Metabolism, 318(3), E330–E342. 10.1152/ajpendo.00272.2019 31846370

