# Supplementary figures and images for: The p21‐activated kinase 2 (PAK2), but not PAK1, regulates contraction‐stimulated skeletal muscle glucose transport
Source: Physiol Rep. 2020 Jun 29;8(12):e14460. doi: 10.14814/phy2.14460 (PMC7322983; doi:10.14814/phy2.14460)

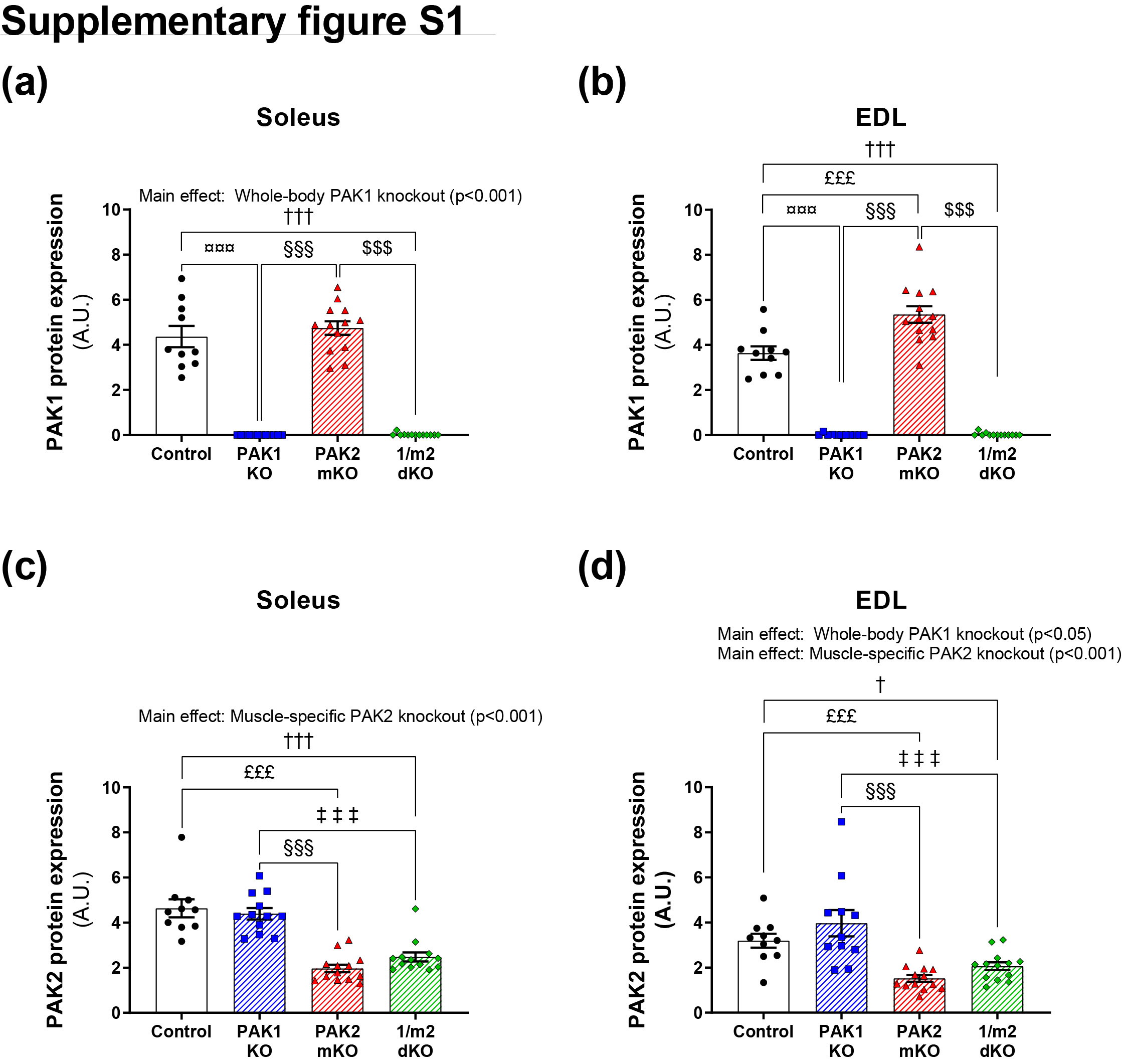

Supplement: Supplementary file 1 — Figure S1 [file PHY2-8-e14460-s001.jpg]
