# Supplementary material for: The p21‐activated kinase 2 (PAK2), but not PAK1, regulates contraction‐stimulated skeletal muscle glucose transport
Source: Physiol Rep. 2020 Jun 29;8(12):e14460. doi: 10.14814/phy2.14460 (PMC7322983; doi:10.14814/phy2.14460)
Supplement: Supplementary file 2 — Caption [file PHY2-8-e14460-s002.docx]

**Supplementary figure S1:** **(a-d)** Quantification of PAK1 and PAK2 protein expression in soleus (a, and c) and extensor digitorum longus (EDL; b and d) muscles from whole-body PAK1 knockout (KO), muscle-specific PAK2 (m)KO, PAK1/2 double KO (1/m2 dKO) mice or control littermates. Total protein expression is an average of the expression in the left and right muscle from the same mouse. Total protein expression was evaluated with a two-way ANOVA to test the factors ‘PAK1’ (PAK1^+/-^ vs. PAK^-/-^) and ‘PAK2’ (PAK2^fl/fl^;MyoD^+/+^ vs. PAK2^fl/fl^;MyoD^iCre/+^) thereby assessing the relative contribution of PAK1 and PAK2. Differences between genotypes (Control vs. PAK1 KO vs. PAK2 mKO vs. 1/m2 dKO) were evaluated with a one-way ANOVA. Significant one-way ANOVA and interactions in two-way ANOVA were evaluated by Tukey’s post hoc test: Control vs. PAK1 KO ¤¤¤ (p<0.001); Control vs. PAK2 mKO £££ (p<0.001); Control vs. 1/m2 dKO †/††† (p<0.05/001); PAK1 KO vs. PAK2 mKO §§§ (p<0.001); PAK1 KO vs. 1/m2 dKO ‡‡‡ (p<0.001); PAK2 mKO vs. 1/m2 dKO $$$ (p<0.001). For total protein expression, the number of determinations in each group: Soleus, *n = 10/12/13/13* (Control / PAK1 KO / PAK2 mKO / 1/m2 dKO); EDL, *n = 10/11/13/13*. Data are presented as mean $\pm$S.E.M. with individual data points shown. A.U., arbitrary units.
